# Mental Health in Medicine: A novel stepped care model in medical psychiatry and the implementation of measurement-based care

**DOI:** 10.1192/j.eurpsy.2024.349

**Published:** 2024-08-27

**Authors:** D. Wiercigroch, S. Wright, N. Bangloy, S. Bush, A. Wai, D. Koshy, L. Thomson, N. Abdel Gawad, S. Abbey, M. Kaye, M. Kelsey, A. Tan, J. Delwo, K. Sheehan

**Affiliations:** ^1^ University Health Network; ^2^Psychiatry, University of Toronto, Toronto, Canada

## Abstract

**Introduction:**

Individuals with co-occurring mental and physical health issues have worse health outcomes in both domains. Integration improves outcomes and aligns with patient preference, but health services tend to be siloed. The Mental Health in Medicine Clinic (MHiM) supports patients receiving inpatient or outpatient medical or surgical care at a tertiary academic hospital in Toronto, Canada. The predominantly virtual clinic has an interdisciplinary team offering services via stepped care, matching patient need with service intensity. Measurement-based care (MBC), the systematic evaluation of patient reported outcomes, was not initially used routinely in the clinic, but its implementation may improve treatment decision-making and may be useful in allocating patients within a stepped care model.

**Objectives:**

1) To describe the stepped care model, referral patterns, diagnoses, and level of care provided since implementation of stepped care. 2) To conduct a quality improvement initiative to implement MBC in the clinic, with a goal of 50% of patients completing at the time of first assessment and prior to discharge from the clinic.

**Methods:**

We reviewed the electronic medical record for referral source, diagnoses, and level of stepped care within the clinic. We conducted semi-structured interviews with stakeholders (clinicians, administrative staff, patients) to explore barriers to implementation of MBC. Interviews were analyzed for themes around barriers and facilitators to MBC. Plan, Do, Study, Act cycles were carried out around change concepts informed by stakeholder interviews and relevant literature.

**Results:**

The MHiM clinic began operations in August 2020. The clinic operated on a physician-only model until March 2022 and then shifted to a stepped care model with an interdisciplinary team. The most frequent referral sources were internal medicine, COVID19 clinics, consultation-liaison psychiatry, red blood cell disorders clinic and cardiology. Since the implementation of stepped care, 250 referrals were assessed. 58% of new referrals were assessed by the psychiatrist, 42% were managed by the NP, and 25% consulted with the social worker. Referrals consisted of trauma and stress-related disorders (32%), depression (21%) or anxiety disorders (20%). Personality, substance use, and psychotic disorders accounted for less than 10% of referrals combined. Some patients did not have any diagnosis (6%). Results from the quality improvement initiative to implement MBC will also be presented.

**Conclusions:**

The MHiM clinic provides an integrated care pathway addressing comorbid mental and physical health conditions. We describe a novel stepped care model and the implementation of MBC. Future directions include ongoing quality improvement of MBC and its integration within the clinic to assess and re-assess service intensity.

**Disclosure of Interest:**

None Declared

